# Hepatitis E virus genotype 3 microbiological surveillance by the Spanish Reference Laboratory: geographic distribution and phylogenetic analysis of subtypes from 2009 to 2019

**DOI:** 10.2807/1560-7917.ES.2022.27.23.2100542

**Published:** 2022-06-09

**Authors:** Milagros Muñoz-Chimeno, Silvia Bartúren, Maira Alejandra García-Lugo, Lucia Morago, Álvaro Rodríguez, Juan Carlos Galán, Alfredo Pérez-Rivilla, Mercedes Rodríguez, Rosario Millán, Manuel del Álamo, Roberto Alonso, Laura Molina, Aitziber Aguinaga, Ana Avellón

**Affiliations:** 1 Hepatitis Unit, National Centre of Microbiology, Instituto de Salud Carlos III, Majadahonda, Madrid, Spain; 2 Servicio de Microbiología, Hospital Universitario Ramón y Cajal and Instituto Ramón y Cajal de Investigación Sanitaria (IRYCIS), Madrid, Spain; 3 CIBERESP, Madrid, Spain; 4 Hospital Universitario 12 de Octubre, Madrid, Spain; 5 Hospital Universitario Central de Asturias, Grupo de Microbiología Traslacional (ISPA) Oviedo, Asturias, Spain; 6 Hospital Universitario Puerta de Hierro-Majadahonda, Madrid, Spain; 7 Hospital Universitario Severo Ochoa, Leganés, Madrid, Spain; 8 Hospital General Universitario Gregorio Marañón, Madrid, Spain; 9 Hospital Universitario de Fuenlabrada, Madrid, Spain; 10 Complejo Hospitalario de Navarra, Pamplona, Navarra, Spain

**Keywords:** Hepatitis E, HEV, genotype 3, subtype, Spain

## Abstract

**Background:**

Hepatitis E virus genotype 3 (HEV-3) is widely distributed throughout Europe, with incidence of infections increasing in many countries. Belgium, Bulgaria, France, Germany, Italy, the Netherlands and the United Kingdom have reported the distribution of HEV-3 subtypes in cohorts of patients with hepatic disease.

**Aim:**

To describe the distribution of the HEV-3 subtypes in Spain at national and autonomous community (AC) levels between 2009 and 2019. The study was also extended to Andorra.

**Methods:**

Of 5,197 samples received by the National Reference Laboratory during the study, 409 were HEV-RNA-positive. Among these, 294 (71.9%) were further typed based on an ORF2 sequence fragment, or, for a subset of 74, based on the full-coding genome sequence.

**Results:**

HEV-3 was detected in 291 samples. The dominant subtype in Spain was HEV-3f (88.3%; 257/291), which occurred in all ACs, with no change in detection level over time. Within this subtype, three subclusters were characterised: HEV-3f-B, HEV-3f-A1 and HEV-3f-A2. The second most common HEV subtype was the recently described HEV-3m (7%; 21/291), with two subclusters identified: HEV-3m-A, which has been known since 2010, and HEV-3m-B, since 2014. The third most encountered subtype was HEV-3c (4.1%; 12/291), with a frequency not increasing over time, unlike observations in some European countries.

**Conclusion:**

The importance of the surveillance of HEV-3 subtype and subcluster circulation is yet to be assessed. This surveillance together with the comprehensive epidemiological characterisation of clinical cases, could support the identification of sources of transmission and the establishment of control measures nationally and internationally.

## Introduction

Hepatitis E virus (HEV) infection makes up a considerable portion of acute hepatic disease. The number of new HEV infections acquired in the European Union/European Economic Area (EU/EEA) is growing [1]. HEV belongs to the *Hepeviridae* family, and its Orthohepevirus A genus includes viruses that infect humans, pigs, boars, rabbits and camels, among others [2]. Eight genotypes have been described so far, five of which (HEV-1, 2, 3, 4 and 7) infect humans [3]. Epidemiology and transmission of HEV follow two distinct patterns with different characteristics. Genotypes 1 and 2 affect only to humans and cause epidemics in lower-middle income countries through faecal–oral transmission. Genotypes 3 and 4 can both cause zoonosis and can be detected in humans and animals worldwide. Domestic pigs and wild boars are the most important animal reservoirs for these genotypes and the main source of human infection after meat consumption [4]. Genotype 7 cases are very rare.

In 2014, HEV-3 variants were classified into two major clades named 3abchij and 3efg [5], which both included several genetic subtypes. A third clade corresponding to the 3ra subtype had, however, been described in 2009, with strains detected in infected rabbits [6]. According to Smith et al. in 2016 [3], the genetic distance between whole genome sequences (WGS) of viruses divides HEV-3 genotypes into subtypes 3a, 3b, 3c, 3d, 3e, 3f, 3g, 3h, 3i and 3j. In 2017 and 2018, two new HEV-3 subtypes were identified: HEV-3k [7] and HEV-3l [8]. In 2020, Smith et al. [9] updated reference sequences, whereby an additional subtype, HEV-3m was proposed. The update was based on previously published sequences from Spain [10] and France [11], however, some sequences still remained unassigned. Recently, an automated partition of a maximum likelihood phylogenetic tree and distance analysis method suggested the existence of several new subtypes, enabling to classify some of the previously unassigned WGS [12].

The distribution of HEV subtypes in cohorts of patients with hepatic disease has, to our knowledge, been studied in only a few European countries: Belgium [13,14], Bulgaria [15], France [11,16], Germany [17,18], Italy [19,20], the Netherlands [21] and the United Kingdom (UK) [5,22]. In the case of Spain, there is limited information about the distribution of HEV-3 subtypes in humans. All HEV subtypes obtained in a retrospective study of patients in 2000–2004 and 2007–2008 [23] and in liver donors [24] were HEV-3f. In Spanish pig populations, several studies have shown that HEV-3 has long been present, and in 2017, subtypes HEV-3f and HEV-3m were detected in two pig liver samples [25]. HEV-3m has also been found in Spanish wild boar [26].

The present work aims to characterise HEV genotypes, subtypes and main subclusters found in Spain from 2009 to 2019 by phylogenetic and p-distance analyses. Data from Andorra are also analysed. The temporal distribution of subtypes/subclusters throughout the study period is also presented, as well as their geographical occurrence in the different autonomous communities (ACs) of Spain, as well as in Andorra. 

## Methods

### Samples

From 2009 to 2019, the National Centre of Microbiology, Instituto de Salud Carlos III, Majadahonda (Madrid), Spain, acting as HEV reference laboratory, received 5,197 serum samples for HEV RNA detection. These originated from public hospitals of all ACs of Spain. Four samples from Andorra were also included. Samples had been sent to the reference laboratory for confirmation of previous positive anti-HEV results or confirmation of primary HEV diagnosis after negative findings for hepatitis A, B and C viruses. HEV-RNA-positive samples were included in the study.

### Ribonucleic acid extraction and retro-transcription

RNA was extracted automatically from 200-µL serum samples with the Magna Pure LC 2.0 System (Roche Diagnostics, Germany) following the total NA protocol. Complementary DNA (cDNA) was obtained by reverse-transcription with random hexamers using a Transcription First Strand cDNA Synthesis kit (Roche Diagnostics, Mannheim, Germany), following the manufacturer’s recommendations.

### Amplification and sequencing

A 459-bp fragment of the HEV open reading frame (ORF)2 region was amplified by nested PCR, using as template a 916-bp amplicon obtained from a primary PCR on the cDNA. For both the primary and nested PCRs, 5 µL of DNA were used in a total 50-µL-reaction volume containing PCR Master Mix and RNase-free water (Promega, Madison, WI, United States (US)). The primers for the primary PCR were 5'-GAGYTGGTYATCCCIAGTGAGCG-3' (forward) and 5'-CCTTRGTCGTRCCAGCYTCCCA-3' (reverse), and the PCR temperature profile comprised 4 min at 94 °C (initial denaturation), then 40 cycles including 1 min at 94 °C (denaturation), 1 min at 57 °C (annealing) and 1 min at 72 °C (elongation), followed by a final elongation for 5 min at 72 °C. For the nested PCR, the primers were 5'-GGTGTSGCYGAGGARGAGGC-3' (forward) and 5’-CCYTTRTCYTGCTGYGCATTCTC-3’ (reverse) and the temperature profile was 2 min at 94 °C (initial denaturation), then 35 cycles including 1 min at 94 °C (denaturation), 1 min at 57 °C (annealing) and 1 min at 72 °C (elongation), followed by a final elongation for 5 min at 72 °C. PCR products were separated by electrophoresis on 2% agarose gels in Tris-borate- ethylenediaminetetraacetic acid (TBE) buffer at a final concentration of 0.5× and stained using Biotium GelRed (Hayward, CA, US).

Amplification products were purified with Illustra ExoProStar 1-STEP (VWR International Eurolab S.L., Radnor, Pennsylvania, US). Sense and antisense DNA strands were both sequenced by the Sanger method.

For 74 clinical serum samples, a full HEV-3 coding genome was respectively obtained through 12 overlapping nested PCRs, mainly as described previously [10].

### Phylogenetic analysis and sequence identification

Sequence alignment, p-distances calculations and phylogenetic trees were derived using the Molecular Evolutionary Genetics Analysis (MEGA) 7.0 package (https://www.megasoftware.net/). A 411-bp fragment (5,960–6,371 according to the sequence with accession number KU513561) was used for ORF2 analysis. The full-coding genomes, excluding the hypervariable region (HVR), ranged from 6,844 to 7,124 bp. Phylogenetic trees were derived by a maximum likelihood (ML) approach using the general time-reversible model (GTR + G + I) according to the substitution model estimated with jModelTest (PhyML 3.0 programme; http://atgc.lirmm.fr/phyml/), based on the Akaike information criterion. Support for tree nodes was assessed by bootstrap values (BT) based on 1,000 replicates.

The reference sequences used were those updated by Smith et al. [9], with the following GenBank accession numbers: M73218 (HEV-1), KX578717 (HEV-2), AF082843 (HEV-3a), AP003430 (HEV-3b), FJ705359 (HEV-3c), AB248521 (HEV-3e), AB369687 (HEV-3f), AF455784 (HEV-3g), JQ013794 (HEV-3h), FJ998008 (HEV-3i), AY115488 (HEV-3j), FJ906895 (HEV-3ra), AB369689 (HEV-3k), JQ953664 (HEV-3l), KU513561 (HEV-3m), AB290313 (HEV-3Mongolia), MF959765 (HEV-3Italy), LC260517 (HEV-3-Japan), MK390971 (HEV-3Italy), MF959764 (HEV-3Italy), KP294371 (HEV-3Germany), AB197673 (HEV-4), AB573435 (HEV-5), AB602441 (HEV-6), KJ496143 (HEV-7), and KX387865 (HEV-8). Sequences obtained in this study were assigned GenBank accession numbers MZ272478–MZ272697 and MZ289076–MZ289149.

### Statistical analysis

Group differences between categories of qualitative variables were investigated with chi-squared tests. Values of p < 0.05 were considered to be significant.

## Results

### Total number of analysed sequences

From the 5,197 total samples received by the HEV reference laboratory, 409 (7.87%) were positive for HEV RNA (Supplementary Table S1, Representativeness of obtained sequences from yearly studies samples). From the latter, 294 sequences were respectively obtained (representing 71.9% from the total RNA positive), with only partial-ORF2 sequences for 220 and full-coding HEV genome sequences for 74. The proportion of HEV sequences analysed by year among the samples testing positive for HEV ranged from one of five in 2009 to 52 of 53 in 2019.

### Assessing full-coding genome vs open reading frame 2 sequences for hepatitis E virus genotype 3 subtyping

**Figure 1:**
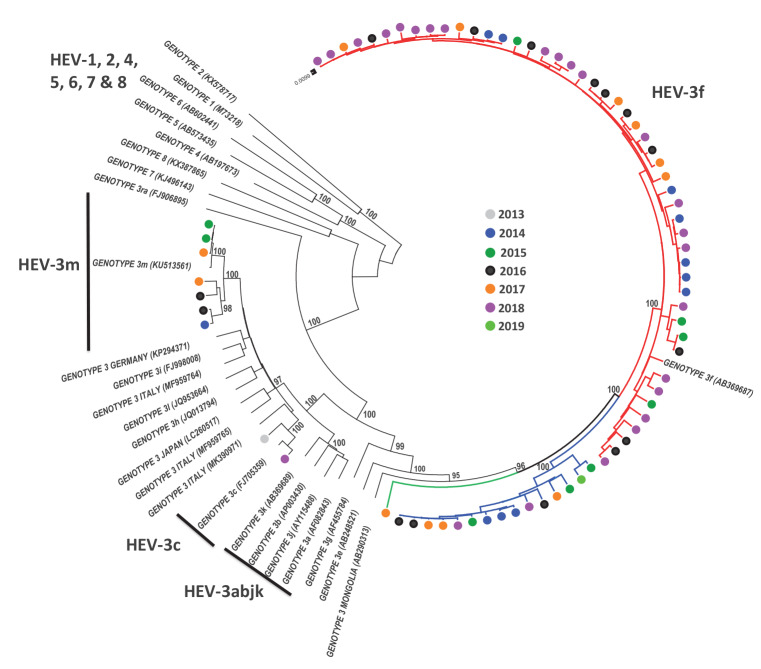
Phylogenetic analysis, using HEV genotype- and subtype-reference sequences, of 74 full-coding genome sequences of HEV-3 from Spain, 2009–2019 (n = 100 total sequences in the tree)

For 74 samples, results of phylogenetic analyses based on partial-ORF2 sequences (411 nt) and based on full-coding-genome sequences were compared to assess whether the partial ORF2 could reliably be used to determine HEV-3 clades, subtypes and subclusters. The topology of the phylogenetic tree with ORF2 sequences (Supplementary Figure S1, Phylogenetic tree of 74 ORF2 fragment sequences) was very similar to that obtained from full-coding-genome sequences (Figure 1), with weaker, though still sufficient, bootstrap support, and similar genetic p-distances to reference sequences in clusters defining subtypes (Supplementary Table S2, Comparison of phylogenetic analysis between full-coding genome and ORF2 411 NT fragment in 74 sequences).

HEV: hepatitis E virus.

Potential HEV-3f-A1, HEV-3f-A2 and HEV-3f-B subclusters are shown by red, blue and green lines, respectively. Sequences obtained in this study are indicated by coloured circles with colour according to year of sampling. Reference sequences of HEV genotypes 1, 2, 3, 4, 5, 6, 7 and 8 were included in the analysis. Genbank accession numbers are indicated on the tree leaves. Bootstrap values indicating the number of repetitions of the tree with the showed topology are included in the tree nodes defining clades, genotypes, subtypes, clusters and subclusters.

### Description of potential subclusters in hepatitis E virus genome 3 subtypes f and m

From the full-coding genome sequence analysis illustrated in Figure 1, we observed potential subclusters within HEV-3f (A1, A2 and B) and HEV-3m (A and B).

**Figure 2:**
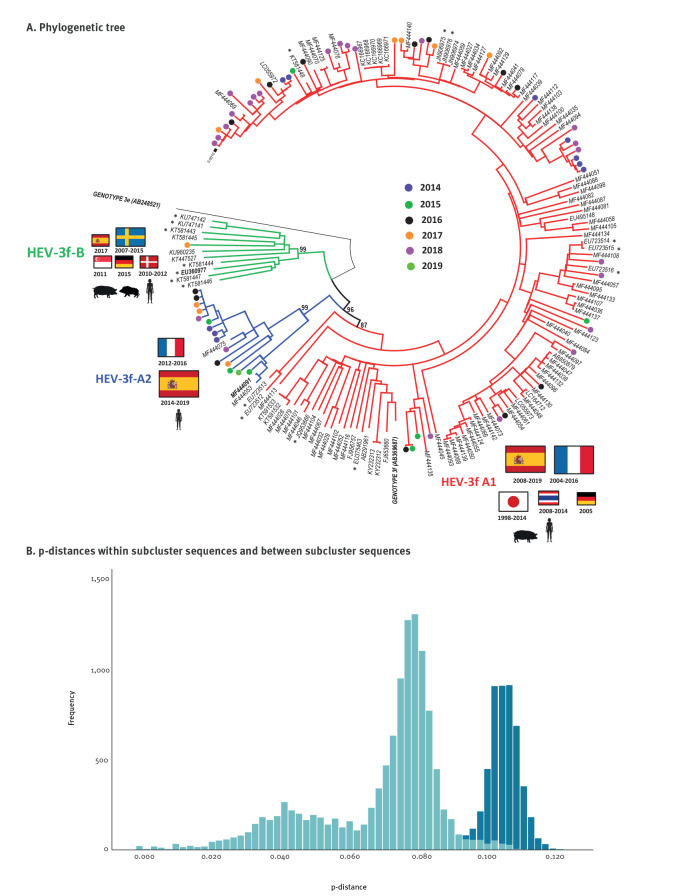
(A) Phylogenetic and (B) p-genetic distance investigations of HEV-3 subtype f subclusters found among full-coding genome sequences recovered from different countries, 2013–2019 (n = 181 sequences)

Focusing first on HEV-3f (Figure 2a), the phylogenetic tree including 181 full-coding genome sequences showed a HEV-3f-A1 subcluster, in which 151 sequences joined the AB369687 reference sequence (BT99), a HEV-3f-A2 subcluster including 18 sequences (BT87) and a HEV-3f-B subcluster with 11 sequences (BT99). A sequence of genotype 3e was used as an outgroup. A histogram of intragroup and intergroup p-distances (Figure 2b) showed minimum overlapping values: the intragroup averages (and ranges) were 0.079 (0.000–0.112), 0.070 (0.004–0.099) and 0.072 (0.025–0.101), for HEV-3f-A1, HEV-3f-A2 and HEV-3f-B, respectively, while the mean intergroup distance was 0.106 (0.091–0.123).

HEV-3: hepatitis E virus genotype 3.

A. Potential HEV-3f-A1, HEV-3f-A2 and HEV-3f-B subclusters are shown by red, blue and green lines, respectively. Countries (flags) and year of detection are shown next to each subcluster name. The sizes of the flags reflect the number of sequences from each country (smallest-sized flag: 1–2 sequences; medium-size flag: 3–7 sequences; largest-size flag: ≥ 8 sequences). Sequences of animal origin are shown with an asterisk. The 65 sequences from Spain analysed in this study are indicated by coloured circles, with colour according to the year of sampling. Genbank accession numbers of sequences used for the phylogenetic analysis are indicated on the tree leaves. Bootstrap values indicating the number of repetitions of the tree with the showed topology are included in the tree nodes defining clades, genotypes, subtypes, clusters and subclusters.

B. Histogram comparing the frequencies of p-distance values in intra-group (light blue) vs inter-group (dark blue) sequence comparisons.

Considering HEV-3m (Supplementary Figure S2, Phylogenetic tree of complete genome HEV-3m sequences), the analysis of 11 full-coding genome sequences revealed some potential subclusters: HEV-3m-A, including five sequences and the KU513561 reference sequence (BT100), and HEV-3m-B, with five sequences (BT99). The average and range of the intra-group p-distances in HEV-3m-A and HEV-3m-B were 0.014 (range: 0.000–0.024) and 0.062 (range: 0.011–0.088), respectively, and the average intergroup distance was 0.095 (range: 0.092–0.099).

For further analyses of HEV-3, we consider EU360977, MF444091 (Figure 2) and MF444030 (Supplementary Figure S2) as reference sequences for potential subclusters of HEV-3f-B, HEV-3f-A2 and HEV-3m-B, respectively, as they were the first to be published in the GenBank database.

### Temporal and geographical distribution of hepatitis E virus genotypes

**Figure 3:**
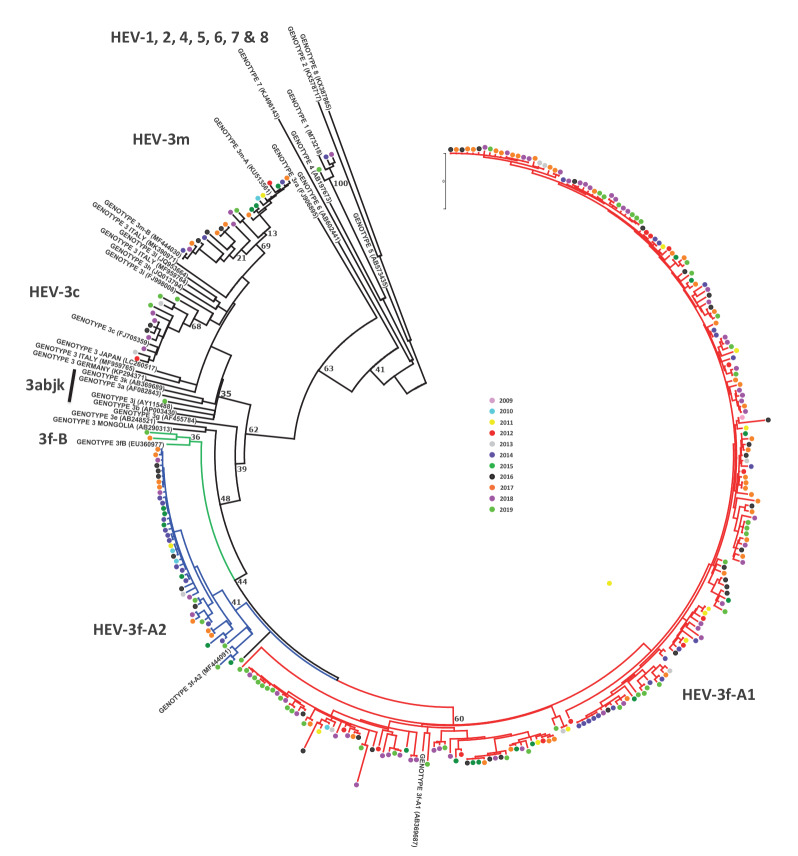
Phylogenetic analysis of 294 ORF2 fragment genetic sequences of HEV recovered in Spain, including two sequences of Andorra, 2013–2019 (n = 323 sequences in the tree)

HEV: hepatitis E virus; ORF2: open reading frame 2.

A total of 294 sequences were genotyped using the 411-bp ORF2 fragment (Figure 3). Three samples detected in 2014, 2018 and 2019 were HEV-1 (subtype 1g) (3/294; 1.0%). The remaining samples were HEV-3 (291/294; 99.0%).

Potential HEV-3f-A1, HEV-3f-A2 and HEV-3f-B subclusters are shown with red, blue and green lines respectively. The 294 sequences obtained in this study are indicated with coloured circles, with colour according to year of sampling. Reference sequences of HEV genotypes 1, 2, 3, 4, 5, 6, 7 and 8 were included in the analysis. Genbank accession numbers of sequences used for the phylogenetic analysis are indicated on the tree leaves. Bootstrap values indicating the number of repetitions of the tree with the showed topology are included in the tree nodes defining clades, genotypes, subtypes, clusters and subclusters.

HEV-3f was the most prevalent subtype (257/291; 88.3%), followed by HEV-3m (21/291; 7.2%) and HEV-3c (12/291; 4.1%). One sequence (MZ272662), found inside the 3abjk clade and obtained in 2019 in Andalusia, did not group with any previously described subtype reference sequence (the closest being HEV-3b, with a p-distance of 0.110).

HEV-3f-A1, HEV-3f-A2 and HEV-3f-B accounted for 212 (72.8%), 42 (14.4%) and two (0.7%) of the 291 HEV-3f positive samples, respectively. One sequence from 2019 (MZ272695) did not group with any of the three described HEV-3f groups (Figure 3), but was closest to HEV-3f-A1, with a p-distance of 0.076.

The temporal and geographical distributions of the subtypes and subclades are shown in Table 1 and Table 2. The analysis of the distribution of subtypes and subclades over time showed that HEV-3m-B was first detected in Spain in 2014 and HEV-3f-B in 2017. No statistical differences were observed for any of the HEV subtypes or subclusters when comparing the periods 2009–2013 and 2014–2019 (p > 0.05).

**Table 1:** Temporal distribution of samples testing positive for HEV-3 according to subtype and subcluster, Spain, 2009–2019 (n = 291 samples)

Subtype	Year											Total 2009–2019		Periods				p value
	2009	2010	2011	2012	2013	2014	2015	2016	2017	2018	2019	n	%	2009–2013		2014–2019		
														n	%	n	%	
HEV-3abjk	0	0	0	0	0	0	0	0	0	0	1	1	0.3	0	0.0	1	0.4	NA
HEV-3c	0	0	0	1	2	0	0	2	0	4	3	12	4.1	3	7.5	9	3.6	0.25
HEV-3f	1	3	11	12	7	30	17	32	46	52	46	257	88.3	34	85.0	223	88.8	0.48
HEV-3f A1	1	1	10	12	6	19	11	26	38	48	41	213	73.2	30	75.0	183	72.9	0.78
HEV-3f A2	0	2	1	0	1	11	6	6	7	4	4	42	14.4	4	10.0	38	15.1	0.39
HEV-3f B	0	0	0	0	0	0	0	0	1	0	1	2	0.7	0	0.0	2	0.8	NA
HEV-3m	0	1	1	1	0	3	3	3	5	3	1	21	7.2	3	7.5	18	7.2	0.94
HEV-3m A	0	1	1	1	0	1	3	0	2	1	1	11	3.8	3	7.5	8	3.2	0.18
HEV-3m B	0	0	0	0	0	2	0	3	3	2	0	10	3.4	0	0.0	10	4.0	NA
**Total**	**1**	**4**	**12**	**14**	**9**	**33**	**20**	**37**	**51**	**59**	**51**	**291**	**100**	**40**	**100**	**251**	**100**	**NA**

HEV-3: hepatitis virus genotype 3; NA: not applicable.

**Table 2:** HEV-3 subtype distribution in different geographical areas, Spain and Andorra, 2009–2019 (n = 289 samples)^a^

Subtype Subcluster	Spanish autonomous communities^b^																	AD	Total		Geographical groups							
	CM	CL	MD	EX	AR	IB	CT	VC	MC	AS	CB	NC	GA	RI	PV	AN	CN											
																					Centre^c^		East^d^		North^e^		South^f^	
																			n	%	n	%	n	%	n	%	n	%
HEV-3abj	0	0	0	0	0	0	0	0	0	0	0	0	0	0	0	1	0	0	1	0.3	0	0.0	0	0.0	0	0.0	1	3.8
HEV-3c	0	1	5	0	0	1	1	2	0	0	0	0	1	0	0	1	0	0	12	4.2	6	4.0	4	12.9	1	1.2	1	3.8
HEV-3f	8	8	109	5	2	3	7	9	4	20	5	14	12	2	22	21	2	2	255	88.2	130	86.1	27	87.1	75	92.6	23	88.5
HEV-3f-A1	6	7	85	4	1	2	7	8	4	18	4	13	12	1	19	17	2	1	211	73.0	102	67.5	23	74.2	67	82.7	19	73.2
HEV-3f-A2	2	1	23	1	1	1	0	1	0	2	0	1	0	1	3	4	0	1	42	14.5	27	17.9	4	12.9	7	8.7	4	15.4
HEV-3f-B	0	0	1	0	0	0	0	0	0	0	1	0	0	0	0	0	0	0	2	0.7	1	0.7	0	0.0	1	1.2	0	0.0
HEV-3m	1	2	11	1	0	0	0	0	0	3	0	2	0	0	0	1	0	0	21	7.3	15	9.9	0	0.0	5	6.2	1	3.8
HEV-3m-A	1	2	3	0	0	0	0	0	0	2	0	2	0	0	0	1	0	0	11	3.8	6	4.0	0	0.0	4	4.9	1	3.8
HEV-3m-B	0	0	8	1	0	0	0	0	0	1	0	0	0	0	0	0	0	0	10	3.5	9	5.9	0	0.0	1	1.2	0	0.0
**Total**	**9**	**11**	**125**	**6**	**2**	**4**	**8**	**11**	**4**	**23**	**5**	**16**	**13**	**2**	**22**	**24**	**2**	**2**	**289**	**100**	**151**	**100**	**31**	**100**	**81**	**100**	**26**	**100**

AD: Andorra.

**^a^** Among 291 samples positive for HEV-3, 289 were accompanied by data on the autonomous community where the patient was located.

^b^ AN: Andalusia; AR: Aragon; AS: Principality of Asturias; CB: Cantabria; CL: Castile and León; CM: Castile-La Mancha; CN: Canary Islands; CT: Catalonia; EX: Extremadura; GA: Galicia; HEV-3: hepatitis virus genotype 3; IB: Balearic Islands; MC: Region of Murcia; MD: Community of Madrid; NC: Chartered Community of Navarre; PV: Basque Country; RI: La Rioja; VC: Valencian Community.

^c^ Centre: CM, CL, MD and EX.

^d^ East: AR, IB, CT, VC and MC. AD data were also included in the ‘East’ group as this country locates to the east of Spain.

^e^ North: AS, CB, NC, GA, RI and PV.

^f^ South: AN and CN.

**Figure 4:**
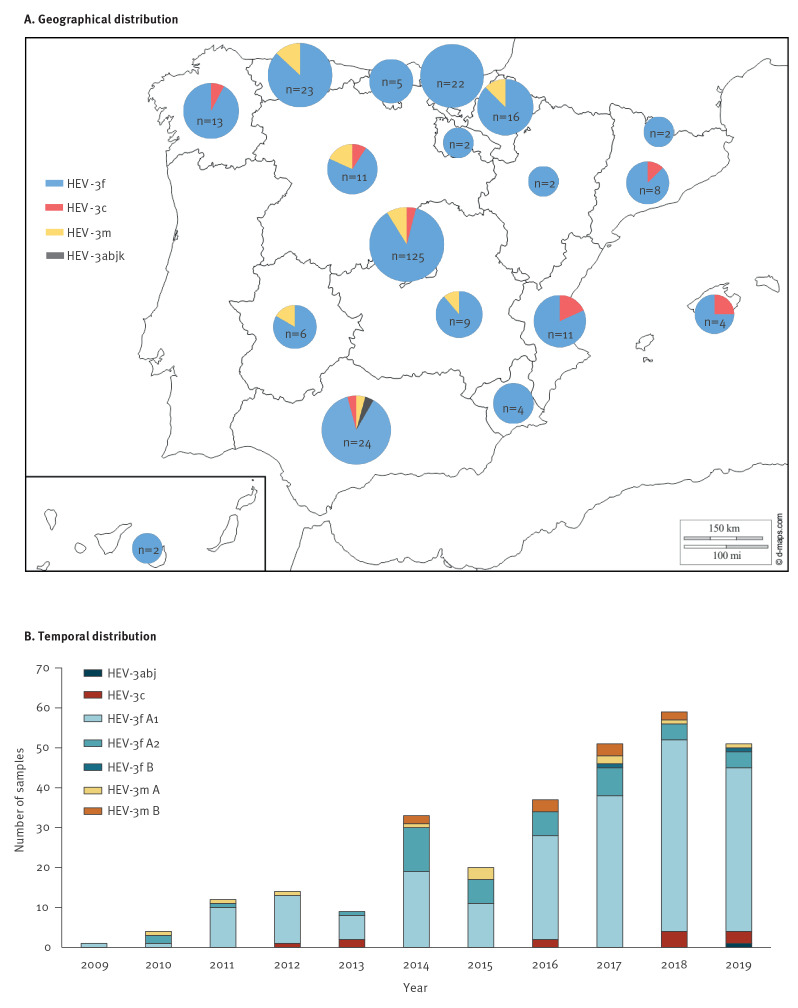
(a) Geographical distribution of samples testing positive for hepatitis E virus genotype 3 according to subtype and (b) temporal distribution with further stratification of subtypes at the subcluster level, Spain and Andorra, 2009–2019 (n = 289 samples)^a^

The geographical location of patients infected with HEV-3 was known for 289 samples (Figure 4 and Table 2). All the ACs of Spain were represented, including the Balearic and Canary Islands. However, no sequences were available from the Autonomous Cities of North Africa (Ceuta and Melilla). Data from Andorra were also included in the dataset with a total of two HEV-3f positive samples over the study period. Examination of the distribution of geographical subtypes showed that the HEV-3f subtype was widely distributed throughout Spain but was most frequent in the north of the country (p = 0.000). HEV-3c was present in some of the ACs, being most prevalent in those in the east (p = 0.005). We found no differences in the distribution of the HEV-3f subclusters. The HEV-3m subtype was reported in the centre, north and south of the country, but no cases were found in the east.

## Discussion

Hepatitis E is not notifiable in Spain and consequently temporal trends of the incidence of cases are not available. Information on HEV from the Spanish National Reference Laboratory is used for microbiological surveillance and has contributed to work assessing the epidemiological situation of HEV in humans in Europe [1]. In the current work, 71.9% of samples testing positive for HEV RNA in the reference laboratory between 2009 and 2019 were further characterised by sequencing, phylogenetic or genetic p-distance analyses. The data obtained were used to assess the temporal and geographical distribution of HEV-3 subtypes in Spain, both nationally and at the AC level.

While 71.9% of HEV-RNA-positive samples were typed, the annual proportions of sequenced positive samples appeared to be higher in the years of prospective investigations (2018 and 2019) than in the earlier years of the study. Moreover, between the beginning and the end of the study period, there seemed to be an increase in the proportions of positive samples sequenced.

Although HEV WGS are obtainable by Sanger sequencing [10,27] or next-generation sequencing (NGS) after enrichment by amplification, WGS are not routinely used for HEV typing for technical reasons. Here, 294 HEV-positive samples were analysed based on the sequence of an ORF2 fragment. To avoid non-congruent subtyping, HEVnet 2019 recommendations to employ a minimum ORF2 sequence length of 300 nt were followed [28], and the ORF2 fragment used was 411 bp long, which is more than that in other European studies [12,15,17,19,21]. Moreover, to check the reliability of this fragment for typing, a subset of 74 study samples was subjected to characterisation both based on the fragment data and based on the full-coding genome sequence data. As the results produced by the two approaches were similar, the fragment-based method was deemed adequate for defining not only subtypes, but also subclusters.

HEV-3f was found to be the dominant subtype in Spain, accounting for 88.3% of HEV-3-typed viruses in the period from 2009 to 2019, with no significant change in frequency during that time. The subtype was present in all Spanish ACs. HEV-3f was likewise the main subtype during a similar period in Belgium (50.7%) [14], south-western France (73.6%) [16] and Italy [19], occurring also, but not as the most prevalent subtype, in Germany, the Netherlands, the UK, and Bulgaria [5,15,17,21]. For Bulgaria, between 2013 and 2015, it was reported that 24% of the subtyped samples of patients with acute hepatitis E corresponded to HEV-3f, while the majority was represented by HEV-3e (62%) [15].

Three subclusters of viruses were identified within HEV-3f in the current study, namely HEV-3f-A1, A2, and B. These are described through phylogenetic and p-distance analyses of full-coding genome sequence. HEV-3f-A1 and HEV-3f-A2 have been present in Spain since 2009–2010. Indeed, HEV-3f-A2 seems to be a mainly Spanish subcluster, with inside, only three non-Spanish sequences, collected from France in 2012, 2015 and 2016 according to the phylogenetic analysis. By contrast, HEV-3f-A1 occurs throughout Europe (Spain, Germany and France) and Asia (Japan and Thailand), where it has been detected in human and pigs. A study from Italy described HEV-3f (typed by us as HEV-3f-A1, data not shown) and HEV-3e as producing outbreaks in this country in 2019 [20]. HEV-3f-B is a well-defined subcluster with sequences derived mainly from pigs in Sweden and Denmark, and it also includes sequences originating from human infections in Germany and Singapore. 

We found the recently described HEV-3m in Spain [9]. This subtype appears to have been circulating in Spain since at least 2010 [10] at a frequency of more than 7% since then, making it the second most common HEV subtype. HEV-3m has been reported occasionally in France, and the UK [5,11,29] in humans, and described in a wild boar in Spain [26]. It has also been suggested that this subtype is associated with the consumption of contaminated meat containing wild boar [30]. Two different subclusters within HEV-3m were identified in the current study: HEV-3m-A, which has been present in Spain since 2010, and HEV-3m-B, which appeared in 2014.

HEV-3c is overall the third most common subtype in Spain (4.1% of cases). In the east (Catalonia, Valencia and the Balearic Islands) and in the north-west (Galicia) of the country however, it is the second most frequent subtype. In the Netherlands, from 2009 to 2013, 68.8% of the HEV-3 cases grouped with HEV-3c compared to 22.2% with HEV-3f [21]. In Belgium, a study conducted from 2010 to 2017 showed that HEV-3c was the second main subtype (39.8%) after HEV-3f (50.7%) [14]. In South-western France, from 2003 to 2014, the HEV-3c subtype (19.9%) was also the second most represented after HEV-3f (73.6%) [16] and, in the same way, data from viruses collected in Italy, from 2000 to 2018 indicated that HEV-3c and HEV-3e followed HEV-3f as the second most common Italian subtypes [19]. 

While in Spain, the prevalence of HEV-3c subtype has not increased over time, a considerable increase of this subtype has been described in Belgium, France, Germany and the UK [5,11,14,17]. A study in France described an increase of HEV-3c from 2005–2010 to 2011–2016 [11]. An increase of the genetic HEV group 2 (the abchij clade, which includes HEV-3c) was described in the UK first in the period from 2003 to 2012 [5], and subsequently, after a retrospective surveillance study in England and Wales from 2008 to 2017 [22]. In Germany, from 2006 to 2007, cases of HEV were predominantly HEV-3f and HEV-3a [18], while HEV-3c predominated from 2009 to 2016 [17]. 

The epidemiological and public health importance of the surveillance of HEV-3 subtype circulation is yet to be assessed. It has been suggested that a change of dominant HEV-3 subtype over time in the UK might be related to the rise of the number of cases due to population susceptibility to new variants [5]. The relative increase in the frequency of cases of a specific subtype might be related to the emergence of variants and the selection of viruses, or to the introduction of a new epidemiological factor, in the case of HEV-3, through a change in the food chain. In this sense, an increase in the number of cases in the UK was reported to be associated with pork products originating from outside the country and thus reflecting pork products related market issues [31]. This highlights the importance of making surveillance and control measures of HEV infection transnational and of closely watching new variants, such as the recently described HEV-3m, which is emerging in Spain and could be a variant of concern in the future.

There are some limitations of this study. First, as it is based on microbiological surveillance instead of epidemiological surveillance, epidemiological data are not available; second, the total coverage of the country might be affected by the fact that some ACs might not be continuously sending samples for HEV RNA typing to the reference laboratory.

Subtype and subcluster surveillance, together with the comprehensive epidemiological characterisation of clinical cases, including detailed information about the food eaten by the patient in the weeks preceding the appearance of the symptoms, could provide a useful tool for food tracking, to identify sources of transmission and to establishing control measures at the Spanish and European levels.

In summary, HEV-3f, present in all Spanish ACs, was the dominant subtype (88.3%) in Spain between 2009 and 2019, with no change in its level of occurrence over time. Three subclusters within this subtype were characterised for the first time in the current study: HEV-3f-B, HEV-3f-A1 and HEV-3f-A2. The recently described HEV-3m was the second most common HEV subtype, circulating in Spain at a frequency of over 7%. Two subclusters were identified: HEV-3m-A, which has been circulating in Spain since 2010, and HEV-3m-B, since 2014. HEV-3c was the third most frequent subtype in Spain (4.1%), and its proportion did not increase over time, contrary to what was observed in some countries in Europe.

## Conclusions

Although the epidemiological and public health importance of the surveillance of HEV-3 subtype circulation is yet to be assessed, changes circulation patterns might be related to the increase of population susceptibility to recently introduced new variants. Surveillance of the subtypes and subclusters, and the comprehensive epidemiological characterisation of clinical cases, could provide a useful tool for food-tracking in these cases, identifying sources of transmission and contributing to the establishment of control measures at the national and European levels.

## Supplementary Data

1500000 Supplementary Material
