# Supplementary material for: Hepatitis E virus genotype 3 microbiological surveillance by the Spanish Reference Laboratory: geographic distribution and phylogenetic analysis of subtypes from 2009 to 2019
Source: Euro Surveill. 2022 Jun 9;27(23):2100542. doi: 10.2807/1560-7917.ES.2022.27.23.2100542 (PMC9198656; doi:10.2807/1560-7917.ES.2022.27.23.2100542)
Supplement: Supplementary Material [file 2100542_SupplementaryMaterial.pdf]

Supplementary Figures and Tables

This supplementary material is hosted by Eurosurveillance as supporting information alongside the article “Hepatitis E virus genotype 3 microbiological surveillance by the Spanish Reference Laboratory: geographic distribution and phylogenetic analysis of subtypes from 2009 to 2019” on behalf of the authors who remain responsible for the accuracy and appropriateness of the content. The same standards for ethics, copyright, attributions and permissions as for the article apply. Supplements are not edited by Eurosurveillance and the journal is not responsible for the maintenance of any links or email addresses provided therein.

1.1 Supplementary Figures

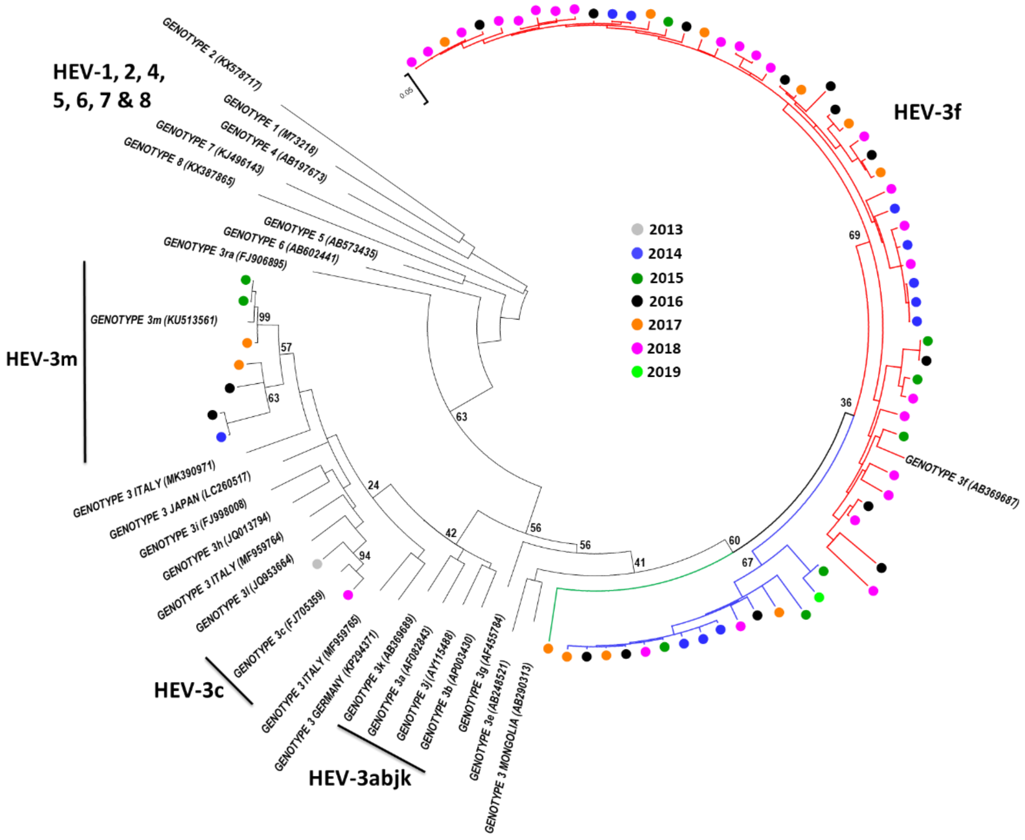

Supplementary Figure S1. Phylogenetic tree of 74 ORF2 fragment (411 bp) sequences. Potential HEV-3f-A1, HEV-3f-A2 and HEV-3f-B sub-clusters are shown with red, blue and green lines,

respectively. Sequences obtained in this study are indicated by colored circles by year. Reference HEV genotypes 1, 2, 3, 4, 5, 6, 7 and 8 sequences were included in the analysis.

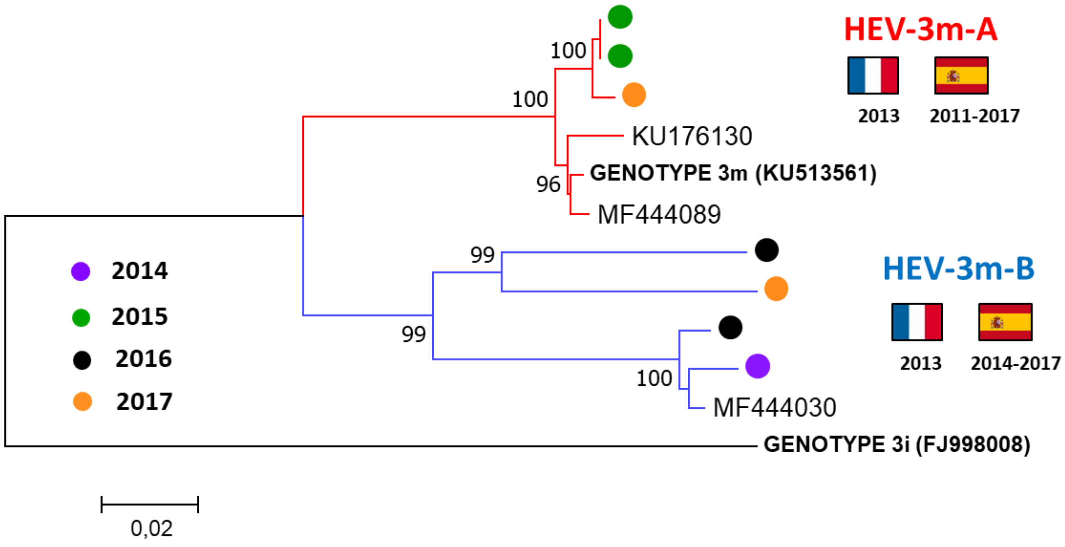

Supplementary Figure S2. Phylogenetic tree of complete genome HEV-3m sequences. Potential HEV-3m-A and HEV-3m-B sub-clusters are shown by red and blue lines, respectively. Countries (flags) and year of detection are shown next to the cluster name, while 7 sequences from Spain analyzed in this study are indicated with circles, coloured by year of detection.

1.2 Supplementary Table

Supplementary Table S1: Representativeness of obtained sequences from yearly studies samples

	2009	2010	2011	2012	2013	2014	2015	2016	2017	2018	2019	Total
Total samples received	124	156	152	231	375	696	715	697	819	790	442	5197
Total HEV RNA Positive	5	12	15	31	18	55	32	57	68	63	53	409
Percentage of HEV RNA positive	4,03	7,69	9,87	13,42	4,80	7,90	4,48	8,18	8,30	7,97	11,99	7,87
Obtained sequences	1	4	12	14	9	34	20	37	51	60	52	294
Percentage of sequences from the total positive	20,00	33,33	80,00	45,16	50,00	61,82	62,50	64,91	75,00	95,24	98,1	71,88

Supplementary Table S2: Comparison of phylogenetic analysis between full coding genome and ORF2 411 NT fragment in 74 sequences. Table shows the number of sequences (N), tree support

(BT=bootstrap value), average p-distance (and range) to reference sequences in both phylogenetic analyses. P-distances have been calculated using FJ705359 for HEV-3c, AB369687 for HEV-3f, and KU513561 for HEV-3m.

	N	Full coding genome		411 NT ORF2 fragment	
		BT	Average p-distance (min-max)	BT	Average p-distance (min-max)
HEV-3f	65	96	0.093 (0.080-0.121)	60	0.098 (0.068-0.137)
HEV-3c	2	100	0.068 (0.041-0.096)	94	0.060 (0.044-0.076)
HEV-3m	7	100	0.058 (0.014-0.093)	57	0.070 (0.020-0.117)
